# New Targeted Therapies and Combinations of Treatments for Cervical, Endometrial, and Ovarian Cancers: A Year in Review

**DOI:** 10.3390/curroncol29040231

**Published:** 2022-04-17

**Authors:** Adelina Silvana Gheorghe, Elena Adriana Dumitrescu, Isabela Anda Komporaly, Raluca Ioana Mihăilă, Cristian Virgil Lungulescu, Dana Lucia Stănculeanu

**Affiliations:** 1 Department of Oncology, “Carol Davila” University of Medicine and Pharmacy, 020021 Bucharest, Romania; elena-adriana.dumitrescu@drd.umfcd.ro (E.A.D.); isabela-anda.komporaly@drd.umfcd.ro (I.A.K.); raluca-ioana.mihaila@drd.umfcd.ro (R.I.M.); dana.stanculeanu@umfcd.ro (D.L.S.); 2 Department of Medical Oncology, University of Medicine and Pharmacy Craiova, 200349 Craiova, Romania; cristilungulescu@yahoo.com

**Keywords:** cervical cancer, endometrial cancer, ovarian cancer, 2021 update, novel targeted therapies, immunotherapy

## Abstract

This review of the meaningful data from 2021 on cervical, endometrial, and ovarian cancers aims to provide an update of the most clinically relevant studies presented at important oncologic congresses during the year (the American Society of Clinical Oncology (ASCO) Annual Meeting, the European Society for Medical Oncology (ESMO) Congress and the Society of Gynecologic Oncology (SGO) Annual Meeting). Despite the underlying existence of the COVID-19 pandemic, the last year has been notable in terms of research, with significant and promising advances in gynecological malignancies. Several major studies reporting the effects of innovative therapies for patients with cervical, endometrial, and ovarian cancers might change the medical practice in the future.

## 1. Introduction

Recent preclinical and clinical research has led to impressive advances in genital cancer, from examining its cellular origins to obtaining an outlook into the mechanisms of DNA damage repair that can be used for various therapies. Moreover, studies have shown clinical benefits for the inhibition of PARP (poly (ADP-ribose) polymerase) and cell cycle modulation and have identified molecular features related to the therapeutic response.

In 2020, the COVID-19 pandemic dominated the medical world, leading to public health policies and scientific research efforts. Nevertheless, for women whose lives are affected by gynecological cancers (mainly cervical, uterine, and ovarian cancers), the impact of these neoplasms on incidence and mortality was taken into account. Thus, clinical care and research were forced to adapt in response to the pandemic, and encouragingly, research in gynecological cancers has remained active.

## 2. Cervical Cancer

Cervical cancer remains one of the most common diagnoses of cancer in women, despite the spread of screening programs. Worldwide, it has a higher incidence and mortality rate than uterine and ovarian cancer, according to Globocan 2020 data, being at fourth place in the incidence in women (604,127 cases detected in 2020, cumulative risk of 1.8%), after breast, colorectal, and lung cancer [1].

### 2.1. Screening and Prevention

In July 2021, the World Health Organization developed and published the second edition of the guide for screening and treatment of pre-cancerous cervical lesions for the prevention of cervical cancer [2]. In addition to vaccination against human papillomavirus (HPV—Human papillomavirus), the main etiological factor for the development of cervical cancer, the implementation of a global screening strategy, could prevent more than 62 million deaths caused by cervical cancer in the next 100 years. The WHO recommends HPV DNA detection in a “screening, triage and treatment” approach from the age of 30, with regular screening every 5 to 10 years, for the general population and from the age of 25, with regular screening every 3 to 5 years, for the HIV-positive population. A recently published study showed that the COVID-19 pandemic dramatically reduced (by 84%) cervical cancer screening in the United States during April 2020 compared with the previous 5-year averages for that month [3].

The results of a recent genomic-wide association study (GWAS) provide new evidence of genetic susceptibility to cervical cancer, especially gene variants of PAX8, CLPTM1L, and HLA, suggesting that their mutations disrupt the pathways of apoptotic and immune functions. Future studies that integrate the interaction between the host and the virus, genetics, and epigenetics, could further elucidate the complex interactions that predispose women to cervical cancer [4].

### 2.2. Surgery

Regarding surgery, minimally invasive radical hysterectomy was associated with lower rates of disease-free survival and OS than open abdominal radical hysterectomy in women with early-stage cervical cancer, according to the LACC study (The Laparoscopic Approach to Cervical Cancer) [5]. After the publication of this study, a recent assessment shows that the use of minimally invasive surgery decreased by 73% in academic centers and by 19% in non-academic centers (*p* = 0.004) [6].

### 2.3. Radiotherapy, Chemotherapy, and Brachytherapy

For locally advanced cervical cancer, radiotherapy concomitant with chemotherapy (cisplatin) and brachytherapy have been the standard of care since 1999. However, many patients relapse, and, in many cases, distant metastases occur. Over time, various studies have suggested that more adjuvant chemotherapy afterward could bring additional benefits. Despite the flaws of these studies—including short follow-up and treatment intolerance—they have changed medical practice in some centers.

The International phase III OUTBACK study tested the effect of four cycles of adjuvant chemotherapy (carboplatin and paclitaxel) after concomitant radio-chemotherapy in women with locally advanced disease (FIGO stages IB1 with positive lymph nodes, IB2, II, IIIB, or IVA), the primary goal being overall survival (OS) at 5 years [7]. After a median follow-up of 5 years, OS at 5 years was 71% in the group with radio-chemotherapy and 72% in the one with the addition of adjuvant chemotherapy (HR = 0.90; *p* = 0.8). The progression-free survival of the disease (PFS) was also similar between arms: 61% and 63%, respectively (HR = 0.86; *p* = 0.6). The study concluded that in women with locally advanced cervical cancer, the adjuvant chemotherapy does not add any benefit to standard concomitant radio-chemotherapy based on cisplatin, as reported at the annual ASCO 2021 conference. In the phase III INTERLACE study, additional induction chemotherapy is evaluated before radio-chemotherapy, which may induce a better response and increased tolerance from the patients [8].

The chemotherapy and the image-guided adaptive brachytherapy (IGABT) based on MRI (magnetic resonance imaging) resulted in effective and long-term stable local control at all stages of locally advanced cervical cancer with tolerable side effects. These results, published in 2021 (prospective EMBRACE-I cohort study), are a positive discovery in the treatment of locally advanced cervical cancer, which could be used as a benchmark for clinical practice and all future studies. At a median follow-up of 51 months, overall 5-year disease control was 92%, being different depending on the FIGO stage: it ranged from 89% in IIA2 and IVB to 98% in IB1 and 100% in IIIA [9].

The final analysis of the PARCER study showed that the incidence of late gastrointestinal toxicity of grade ≥2 was 21.1% with radiotherapy with image-guided intensity-modulated radiotherapy (IG-IMRT), at the end of 3 years, compared to 42.4% with 3D conformal radiotherapy (3D-CRT). However, there were no differences in disease outcomes, as the 3-year pelvic relapse-free survival and disease-free survival in the IG-IMRT versus the 3D-CRT arm were 81.8% versus 84% and 76.9% versus 81.2% [10].

Endostar, the recombinant human (rh)-endostatin (a fragment derived from type XVIII collagen) with anti-angiogenic properties, was analyzed in combination with platinum-based chemotherapy in the first-line treatment of recurrent/metastatic cervical cancer through a single-arm, prospective phase II study. With a median PFS of 12 months, an overall response rate of 50.0%, and a disease control rate of 71.4%, the combination of platinum-based chemotherapy and endostar resulted in a high level of effectiveness [11].

### 2.4. Immunotherapy

Encouraging data have become clear for checkpoint inhibitors as a second-line treatment for recurrent disease. The PD-L1 inhibitor cemiplimab-rwlc became the first immunotherapeutic agent to produce a statistically and clinically significant survival benefit in recurrent or metastatic cervical cancer that progressed after first-line platinum-based chemotherapy. Second-line treatment with cemiplimab resulted in a 27% decrease in the risk of death from chemotherapy in the squamous cell carcinoma population in the global phase III randomized study EMPOWER-Cervical 1/GOG-3016/ENGOT-cx9. The median OS in this group was 11.1 months with cemiplimab compared with 8.8 months with chemotherapy (HR = 0.73; *p* = 0.00306) [12].

FDA (The Food and Drug Administration) has accepted a license application for accelerated approval (application for permission to place a biological product on the market) for balstilimab, an anti-PD-1 antibody, for the treatment of patients with recurrent or metastatic cervical cancer with the progression of disease during or after chemotherapy. Balstilimab is a fully-humanized G4 monoclonal immunoglobulin (IgG4), designed to block PD-1 interaction with its ligands, PD-L1 and PD-L2 [13].

Balstilimab is currently being investigated in clinical trials as monotherapy and in combination with the anti-CTLA-4 antibody zalifrelimab. The findings of a large (155 patients) single-arm phase II study evaluating the safety and antitumor activity of balstilimab in combination with zalifrelimab for up to 2 years in previously treated patients with recurrent/metastatic cervical cancer showed impressive response rates (including complete remissions—8.8%), duration of response (9.3 months—not reached), and OS (69% at 6 months and 52.7% at 12 months), with manageable tolerability. The clinical benefit was highest in patients with PD-L1 positive tumors, but activity was present also in PD-L1 negative tumors [14].

Regardless of PD-L1 expression or concurrent bevacizumab usage, pembrolizumab plus chemotherapy improved PFS and OS in patients with persistent, recurrent, or metastatic cervical cancer, according to the randomized, double-blind, phase III KEYNOTE-826 study, presented at ESMO 2021 as the first interim analysis. These findings show that pembrolizumab plus chemotherapy, with or without bevacizumab may be a new standard of care for this population, with a tolerable safety profile [15].

In patients with recurrent/advanced cervical cancer, toripalimab (a humanized IgG4 antibody specific for human PD-1 receptor), in combination with concurrent chemoradiotherapy, showed promising anti-tumor effectiveness in a retrospective study presented at ESMO 2021: out of 25 patients included, 23 patients had objective responses (16 complete responses and 7 partial responses), with a 6-month duration of response rate of 92%. Moreover, toripalimab had a tolerable safety profile, suggesting that it might be a potential therapeutic option for this population [16].

Tremelimumab (fully human monoclonal antibody against CTLA-4) plus durvalumab (anti-PD-L1 antibody) combined with metronomic oral vinorelbine in recurrent cervical cancer was investigated in the multi-cohort phase I/II MOVIE trial. Phase II of the study met its primary endpoint, the clinical benefit rate: the objective response rate was 41.4% with five complete responses, seven partial responses, and four stable diseases ≥24 weeks. There is further research required for the combination of chemotherapy and immunotherapy in this group of patients [17].

SHR-1701, a new bifunctional fusion protein comprised of a monoclonal antibody against PD-L1 linked to the extracellular domain of TGF-β receptor II, was evaluated in a phase I study for patients with advanced cervical cancer who had progressed on one or two lines of platinum-based therapy (or were intolerant to it). Even though the median PFS was only 1.8 months, SHR-1701 holds promising antitumor activity and may prove to be a treatment option after further research [18].

For many cancers, the combination of antiangiogenic therapy and immune checkpoint inhibitors has emerged as a viable treatment option. A phase II study was carried out to determine if anlotinib (a new multi-target tyrosine kinase inhibitor) combined with sintilimab (a PD-1 antibody) can improve the effectiveness and safety of patients with advanced cervical cancer. In the cohort of 42 patients enrolled, the overall response rate was 61.5% and the disease control rate was 94.9%, with a median PFS of 9.4 months, providing a good perspective of this treatment [19].

Camrelizumab (an anti-PD-1 antibody), apatinib/rivoceranib (tyrosine kinase inhibitor, blocker of vascular endothelial growth factor receptor-2), and albumin-bound paclitaxel (nab-paclitaxel) were assessed in advanced cervical cancer, proving a good interaction in terms of effectiveness, with manageable adverse reactions: overall response rate was 71%, with five complete responses, median PFS was 15.0 months, while the median duration of response and median OS was not reached [20].

### 2.5. Antibody-Drug Conjugates and Vaccines

In a recently published phase II study (innovaTV 204/GOG-3023/ENGOT-cx6), the authors found that tisotumab vedotin (a tissue factor-directed antibody-drug conjugate) produced lasting responses in patients previously treated with recurrent or metastatic cervical cancer [21]. In the study, 101 patients with recurrent or metastatic cervical cancer (squamous cell, adenocarcinoma, or adenosquamous carcinoma) were enrolled between June 2018 and April 2019. The primary goal was the objective response rate, with a median follow-up at the time of the 10-month analysis. Objective response was observed in 24 patients (24%, 95% CI = 16–33%), including a complete response in 7 (7%). Another 49 patients (49%) had stable disease, resulting in a disease control rate of 72%. The average duration of response was 8.3 months, median PFS was 4.2 months, and median OS was 12.1 months [21].

The ENGOT-Cx8/GOG-3024/innovaTV 205 study, reported as interim results at ESMO 2021, showed that both first-line tisotumab vedotin + carboplatin (55% objective response rate, 6% complete responses, and 48% partial responses, median PFS of 6.9 months) and second/third-line tisotumab vedotin + pembrolizumab (35% objective response rate, 6% complete responses, and 29% partial responses, median PFS of 5.6 months) had a promising antitumor activity with acceptable safety profiles in patients with recurrent or metastatic cervical cancer [22].

The interim results of a Korean phase II study indicated the effectiveness of the combination of pembrolizumab with the GX-188E therapeutic DNA vaccine (tirvalimogen teraplasmid) in patients with advanced cervical cancer, HPV-16 or HPV-18 positive. The combination of pembrolizumab and GX-188E (which induces HPV E6- and E7-specific T-cell activation) had a response in 42% of the patients evaluated; 15% had a complete response and 27% had a partial response. Treatment-related adverse events were easily manageable [23].

### 2.6. Targeted Therapy

BUL719 (alpelisib) was used in the treatment of PIK3CA-mutated advanced/recurrent cervical cancer where at least two lines of therapy have failed, in a small study from Istituto Nazionale dei Tumori (Milano, Italy). For the six patients included, the objective response rate was 33% but the disease control rate was 100%, with a mean duration of response of 6.6 months (two patients had a partial response and four patients had stable disease). More research is needed to determine alpelisib’s role in terms of efficacy and safety in PIK3CA-mutated advanced/recurrent cervical cancer [24].

## 3. Endometrial Cancer

Endometrial cancer ranks sixth in incidence in women worldwide, according to GLOBOCAN 2020 data, with 417,367 new cases in 2020 and a cumulative risk of 1.6% [1].

### 3.1. Surgery

Although primary debulking surgery is often considered standard for the treatment of stage IV endometrial cancer, this is associated with significant morbidity and low survival. Neoadjuvant chemotherapy (NACT) was proposed as an alternative treatment strategy. In a cohort study of 4890 women with metastatic endometrial cancer, 952 women (19.5%) were treated with neoadjuvant chemotherapy. Survival for women treated with neoadjuvant chemotherapy was superior to that of women treated with primary debulking surgery for 3 to 8 months after the initiation of treatment, after which survival was superior for those treated with primary debulking surgery. In contrast, results suggest that women treated with NACT, particularly if they ultimately undergo surgery, may have superior survival in the short term. Based on these findings, NACT may be appropriate for select patients with advanced uterine serous carcinoma [25].

### 3.2. Immunotherapy ± Targeted Therapy

On 21 July 2021, the FDA approved pembrolizumab in combination with lenvatinib (a multikinase inhibitor of vascular endothelial growth factor receptors 1–3, fibroblast growth factor receptors 1–4, RET, KIT, and platelet-derived growth factor receptor-α) for patients with advanced endometrial carcinoma who do not have microsatellite instability-high (MSI-H) or mismatch repair deficiency of DNA (dMMR), according to the results of the KEYNOTE-775 Study/Study 309. These patients must have progressive disease after any previous systemic therapy and should not be candidates for curative surgery or radiation therapy [26]. For patients with advanced endometrial cancer other than MSI-H or dMMR, the median PFS was 6.6 months (95% CI = 5.6–7.4 months) in patients receiving pembrolizumab/lenvatinib and 3.8 months (95% CI = 3.6–5.0 months) for those receiving chemotherapy of the investigator’s choice (HR = 0.60, 95% CI = 0.50–0.72, *p* < 0.0001). The average OS was 17.4 months (95% CI = 14.2–19.9 months) and 12.0 months (95% CI = 10.8–13.3 months)—HR = 0.68, 95% CI = 0.56–0.84, *p* = 0.0001), respectively. The objective response rate was 30% (95% CI = 26–36%) and 15% (95% CI = 12–19%), *p* < 0.0001), respectively. The average duration of the response was 9.2 months and 5.7 months, respectively, in the two arms [26].

In addition, the FDA accepted a new application for an additional license for review, seeking the approval of pembrolizumab monotherapy for the treatment of patients with advanced endometrial carcinoma MSI-H or dMMR, after the progression of the disease following any previous systemic therapy and who are not candidates for curative surgery or radiation therapy [27]. The application is based on the general response data of the KEYNOTE-158 study, presented at ESMO 2021. Pembrolizumab proved to have a durable overall response rate (48%), with 14% complete responses, improving survival in heavily pretreated patients with advanced MSI-H or dMMR endometrial cancer. This monotherapy also had manageable treatment-related adverse events [28].

PD-1 inhibitor dostarlimab was granted accelerated approval for the treatment of adult patients with recurrent or advanced endometrial cancer, which progressed during or after a previous platinum-based therapeutic regimen [29] by the FDA. The phase I GARNET study showed an objective response rate of 42.3% (95% CI = 30.6–54.6%), with a complete response in 12.7% of patients. At a median follow-up of 14.1 months, the mean duration of response was not reached, with 93.3% of responses being preserved for at least 6 months [30].

The combination of immunotherapy and targeted therapy was also assessed in endometrial cancer. Anlotinib (novel oral tyrosine kinase inhibitor targeting c-kit, fibroblast growth factor receptor, platelet-derived growth factor receptors, and vascular endothelial growth factor receptor) plus sintilimab (anti-PD-1 immunoglobulin G4 monoclonal antibody) was studied in a prospective open-label, single-arm, phase II clinical trial, in patients with recurrent advanced endometrial cancer. The overall response rate was 77.3%, with a disease control rate of 91.7%, and a median PFS not reached. The median time of the first response was 1.5 months (0.7–12.8), showing a promising treatment alternative after more research in the future [31].

## 4. Ovarian Cancer

There were 313,959 new recorded cases of ovarian cancer in women worldwide (eighth position), according to GLOBOCAN 2020 data, with a cumulative risk of 1.18% [1].

In June 2021, ASCO has published a guide based on resources, which provides evidence-based recommendations for the evaluation of women with ovarian masses, as well as guidance on the treatment of epithelial ovarian cancer in regions that do not have adequate resources to provide high-level care [32]. Assessment of symptomatic adult women includes assessment of symptoms, family history, abdominopelvic ultrasound, and dosing of the serum tumor marker CA-125, where possible. Additional imaging is recommended if CT/MRI resources are available. Diagnosis, staging, and/or treatment involve primarily surgery, before which it is necessary to investigate the presence of metastases. Treatment requires histological confirmation; the surgical goal is to stage the disease and perform complete cytoreduction until the absence of residual disease. In first-line therapy, platinum-based chemotherapy is recommended; in advanced stages, patients may receive neoadjuvant chemotherapy. After neoadjuvant chemotherapy, all patients should be assessed for interval debulking surgery (interval debulking surgery). Targeted therapy is not recommended in environments/countries with limited medical conditions. Specialized interventions are resource-dependent, for example, laparoscopy, fertility preservation surgery, genetic testing, and targeted therapy. Multidisciplinary care for ovarian cancer and palliative care should be provided, regardless of the environment or resources [32].

### 4.1. Surgery

On 29 November 2021, the FDA authorized an adjuvant for the interoperative detection of malignant lesions in adult patients with ovarian cancer [33]. Pafolacianine sodium injection (OTL38) is a fluorescent medication that operates by targeting the folate receptor, which is overexpressed in ovarian cancer, with the aid of near-infrared fluorescence (NIRF) imaging. The main objective was to achieve R0, which is known to be the strongest predictor of overall survival and was supported by the results of a single-arm, multicenter, open-label trial (NCT03180307), in which NIRF imaging with pafolacianine sodium identified extra lesions that were not scheduled for excision and were not discovered by standard white light or palpation in 33% of patients (36 of 109) [34].

### 4.2. Chemotherapy and Anti-Angiogenic Treatment

The first-line therapeutic standard for epithelial ovarian cancer remained the combination of paclitaxel and carboplatin, along with cytoreductive surgery. Maintenance with bevacizumab has been approved since 2016, and more recently, “front-line” maintenance treatment with PARP inhibitors has become the standard of care in ovarian cancer.

The studies GOG-218 and ICON7/AGO-OVAR 11 showed that early and continuous addition of bevacizumab for 15 months and 12 months to the carboplatin/paclitaxel standard, respectively, significantly improved the PFS of the disease. In both studies, the maximum benefit was seen at the time of the highest cumulative exposure of bevacizumab—immediately after the last cycle of bevacizumab [35]. Nonetheless, the optimal duration of bevacizumab has never been clearly established, therefore, the recent randomized phase III ENGOT/GCIG study examined whether prolonging bevacizumab treatment up to 30 months would improve its efficiency [36]. Treatment with bevacizumab for a longer period of time did not improve either PFS or OS in patients with epithelial ovarian cancer, fallopian tubes, or primary peritoneal cancer. Therefore, a bevacizumab treatment duration of 15 months remains the standard of care.

Adding bevacizumab to ixabepilone (azaepothilone B), a microtubule stabilizer, could be a promising treatment strategy for a group of platinum-resistant or refractory ovarian cancer patients, who currently lack a wide range of treatment options, according to data presented at the virtual edition of the annual meeting of the Society of Gynecological Oncology (SGO) 2021. The combination of bevacizumab plus ixabepilone significantly improved the objective response rate, PFS, and OS compared to ixabepilone alone. The results of the randomized phase II study showed that 33% of patients responded to bevacizumab plus ixabepilone compared to 8% of those receiving ixabepilone alone, and median PFS doubled with the combination (5.5 vs. 2.2 months; HR = 0.33), while median OS improved (10.0 vs. 6.0 months; HR = 0.52) [37].

### 4.3. Antibody-Drug Conjugates

In patients with recurrent ovarian cancer, the antibody-drug conjugate mirvetuximab soravtansine co-administered with bevacizumab, has shown anti-tumor activity that leads to lasting responses in platinum “agnostic” cases (resistant/sensitive), with strong folate receptor alpha expression (FR-α) [38]. The combination led to a response rate of 64%, a mean response time of 11.8 months, and a median PFS of 10.6 months in patients with high FR-α expression in the phase I study FORWARD II [38].

The FDA approved the accelerated review for STRO-002 in August 2021, an antibody-drug conjugate anti-FR-α, for the treatment of patients with epithelial ovarian cancer, fallopian tubes, or primary peritoneal cancer resistant to platinum, who have received one to three previous lines of systemic therapy, according to data from the phase I study STRO-001-GM1 [39].

### 4.4. Immunotherapy

The phase III study IMagyn050/GOG 3015/ENGOT-OV39 showed that the addition of atezolizumab to bevacizumab and chemotherapy did not significantly improve PFS in newly diagnosed stage III or IV ovarian cancer patients, neither among all patients nor among those with positive PD-L1 expression [40]. Current evidence does not support the use of immune checkpoint inhibitors in newly diagnosed ovarian cancer.

The phase III study “JAVELIN Ovarian 100” (NCT02718417) was suspended because it did not show any benefit of PFS to the concomitant addition of avelumab and/or as a chemotherapy maintenance treatment (carboplatin/paclitaxel) in patients previously untreated with advanced epithelial ovarian cancer [41]. In addition, the phase III study “JAVELIN Ovarian 200” did not show any significant improvement in PFS or OS with avelumab alone or in combination with pegylated liposomal doxorubicin (PLD) vs. PLD alone in patients with flat or refractory ovarian cancer [42]. These results do not support the use of avelumab in the frontline treatment setting.

Immunotherapy was also studied in the neoadjuvant setting for unresectable stage IIIC/IV ovarian cancer, in the phase Ib INEOV trial. It was proven to be feasible and safe for the administration of neoadjuvant durvalumab +/− tremelimumab with carboplatin and paclitaxel, prior to interval debulking surgery. However, further research is required on this topic [43].

### 4.5. Targeted Therapy

In a phase II study, it was found that the addition of the oral inhibitor Wee1 kinase (adavosertib) to gemcitabine improved PFS and OS in platinum-resistant or refractory patients, with recurrent high-grade serous ovarian cancer. PFS was longer with adavosertib plus gemcitabine (4.6 months [95% CI = 3.6–6.4]) vs. 3.0 months (95% CI = 1.8–3.8) with placebo plus gemcitabine—HR = 0.55, 95% CI = 0.35–0.90. *p* = 0.015) [44].

The novel multi-target tyrosine kinase inhibitor anlotinib was assessed for safety and effectiveness as monotherapy in patients with ovarian cancer that is recurrent or resistant, in a phase II prospective, single-arm, and single-center clinical study. For the 31 patients included, the median PFS was 5.32 months, while the median OS was not reached, with an overall response rate of 25.9% [45]. Anlotinib was also studied in combination with pemetrexed in patients with ovarian cancer resistant to platinum, in a single-arm, open-label, phase II study, showing a median PFS of 9.3 months (95% CI = 5.5–13.2), an objective response rate of 36.4% (95% CI = 17.2–59.3), and a disease control rate of 100.0% (95% CI = 73.5–100) [46].

The interim results of a study designed by adding a plasmid encoding p62/SQSTM1 (a multi-domain protein that regulates inflammation, apoptosis, and autophagy) to the standard gemcitabine chemotherapy proved that it may be effective for patients with platinum-resistant ovarian cancer, resulting in a PFS of 5.7 months (compared to 2.4 months in the control group, *p* = 0.08) [47].

Relacorilant, a selective glucocorticoid receptor modulator, is studied for its capacity to restore sensitivity to chemotherapy. In a tree-arm, randomized, open-label, phase II trial on patients with recurrent platinum-resistant ovarian cancer or platinum-refractory ovarian cancer, relacorilant was assessed in combination with nab-paclitaxel. The study demonstrated that an intermittent regimen of relacorilant the day before, of, and after the administration of nab-paclitaxel (on days 1, 8, and 15, of a 28-day cycle) led to an improved PFS and duration of response. At a median follow-up of 11.07 months, the intermittent regimen significantly improved median PFS compared to nab-paclitaxel alone, 5.55 versus 3.76 months (95% CI = 0.44–0.98) [48].

### 4.6. PARP Inhibitors

After the initial results were extremely positive, at the 5-year follow-up of the pivotal SOLO-1 study in women with newly diagnosed advanced ovarian cancer and BRCA1/2 mutation, the maintenance treatment with olaparib led to a doubling of the PFS, statistically significant, according to data presented at the SGO 2021 Annual Meeting. Median PFS for the general population was maintained well beyond the end of the treatment: 56.0 months with olaparib versus 13.8 months with placebo (HR = 0.33; 95% CI = 0.25–0.43). The 5-year PFS was 48% and 21%, respectively [49].

The phase III SOLO2/ENGOT-Ov21 study showed a numerically but statistically insignificant improvement in the overall goal of survival with olaparib maintenance therapy compared to the placebo in patients with recurrent platinum-sensitive ovarian cancer and a BRCA1/2 mutation (51.7 months vs. 38.8 months) [50].

The randomized phase II trial OCTOVA aimed to compare olaparib with weekly paclitaxel and the combination of olaparib plus cediranib in recurrent ovarian cancer, either after previous PARP inhibitors administration, or anti-angiogenic treatment. The combination of olaparib + cediranib had a higher PFS compared to olaparib in monotherapy (HR = 0.70; 60% CI: 0.57, 0.86; *p* = 0.08). However, there was no difference in terms of PFS between the cohorts that received olaparib and weekly paclitaxel (HR = 0.97, 60% CI: 0.79, 1.19; *p* = 0.55) [51]. The addition of niraparib maintenance treatment after platinum-based first-line chemotherapy with bevacizumab has shown a clinical benefit in patients with advanced ovarian cancer, according to data from the OVARIO study, presented at SGO 2021 [52]. The analysis of the phase II study by OVARIO showed that 62% of patients in the general population remained progression-free at 18 months, including 76% of patients in the homologous recombination deficit (HRD) subgroup and 47% of patients in the homologous recombinant proficiency (HRP) subgroup [53]. In patients with positive, advanced, relapsed BRCA ovarian cancer, the treatment with the PARP inhibitor rucaparib led to a significant improvement in PFS, compared to standard chemotherapy, according to the results of the international phase III study ARIEL4 (7.4 months vs. 5.7 months—HR = 0.64, *p* = 0.001) [54].

A new PARP inhibitor may soon join the treatment of ovarian cancer, according to the data presented at SGO 2021 [55]. The results of the phase III study (NCT03863860) of fuzuloparib (previously called fluzoparib) as maintenance therapy in patients with recurrent platinum-sensitive ovarian cancer showed a 7.4-month improvement in median PFS (12.9 vs. 5.5 months; *p* < 0.0001) and a 75.5% reduced risk of disease progression or death compared to the placebo (HR = 0.25). The investigator-assessed objective response rate was 70.8% (95% CI = 61.5–79.0), while the median investigator-assessed progression-free survival was 10.3 months (95% CI = 9.2–12.0), and the 12-month survival rate was 93.7% (95% CI = 87.2–96.9) [56].

In the ANNIE multicentre, single-arm, phase II trial, the safety and efficacy of niraparib combined with anlotinib were evaluated in patients with platinum-resistant recurrent ovarian epithelial, fallopian tube, or primary peritoneal cancer. The overall response rate was 48.0% (95% CI = 27.0–69.0%, 12 patients with partial responses, 12 with stable disease), while median PFS and median duration of response were not reached, therefore presenting antitumor activity that appears to be promising, but with a hand–foot skin reaction as a treatment-related side event (in 47.5% of patients) [57].

## 5. Conclusions

Despite the pandemic caused by COVID-19, the results presented here show the many therapeutic advances made in 2021 in the field of gynecological cancers (cervical, endometrial, and ovarian). Table 1 summarizes the FDA approvals in gynecological cancers in 2021 [58].

Translational research, focused on the results of preclinical studies, will further lead to the clinical integration of information obtained in the laboratory, in phase II and III studies, establishing an important basis and key research priorities for the future. By continuing the quest for the best treatments by targeting novel and exploitable genetic and biologic abnormalities in cervical, endometrial, and ovarian malignancies, oncologists must be prepared to confront the challenge of achieving clinically substantial improvements in gynecologic oncology patients’ outcomes.

## Figures and Tables

**Table 1:** FDA approvals in gynecological cancers in 2021.

Date	Active Ingredient and Drug Name	FDA-Approved Use
22 April	Dostarlimab-gxly (Jemperli)	Endometrial Cancer
21 July	Pembrolizumab (Keytruda) plus Lenvatinib (Lenvima)	Advanced endometrial carcinoma that is not MSI-H or dMMR.
20 September	Tisotumab Vedotin-tftv	Recurrent or metastatic cervical cancer who experienced disease progression on or after chemotherapy.
13 October	Pembrolizumab (Keytruda) in combination with chemotherapy, with or without bevacizumab (Avastin)	Persistent, recurrent, or metastatic cervical cancer whose tumors express PD-L1 (combined positive score [CPS] ≥ 1), as determined by an FDA-approved test.
	Pembrolizumab (Keytruda) as a single agent	Recurrent or metastatic cervical cancer with disease progression on or after chemotherapy whose tumors express PD-L1 (CPS ≥ 1), as determined by an FDA-approved test.
29 November	Pafolacianine (Cytalux)	Ovarian cancer (to help identify cancerous lesions during surgery)
